# Surufatinib-induced renal thrombotic microangiopathy: first case report and review of literature

**DOI:** 10.1007/s00428-023-03545-2

**Published:** 2023-04-26

**Authors:** Wenjiao Zhu, Wei Wang, Yuanping Shi, Bo Shen, Yan Li

**Affiliations:** 1 Department of Endocrinology, Shanghai Ninth People’s Hospital, Shanghai Jiao Tong University School of Medicine, Shanghai, China; 2 Department of Neurosurgery, Shanghai Ninth People’s Hospital, Shanghai Jiao Tong University School of Medicine, Shanghai, China; 3 Department of Nephrology, Shanghai Ninth People’s Hospital, Shanghai Jiao Tong University School of Medicine, 639 Zhizaoju Road, Shanghai, China

**Keywords:** Surufatinib, Tyrosine kinase inhibitors, Nephrotoxicity, Nephrotic syndrome, Thrombotic microangiopathy

## Abstract

Angiogenesis inhibitors such as tyrosine kinase inhibitors (TKIs) are common therapeutics currently used to treat oncologic disease. Surufatinib is a novel, small-molecule multiple receptor TKI approved by the National Medical Products Administration (NMPA) for the treatment of progressive, advanced, and well-differentiated pancreatic and extrapancreatic neuroendocrine tumours (NETs). Thrombotic microangiopathy (TMA) is a well-documented complication of TKIs targeting the VEGF-A/VEGFR2 signalling pathway. Here, we describe a 43-year-old female patient with biopsy-proven TMA and nephrotic syndrome due to surufatinib treatment for adenoid cystic carcinoma. Histological lesions included glomerular endothelial swelling, widening of subendothelial spaces, mesangiolysis, and double contour, which caused nephrotic proteinuria. Effective management was achieved by drug withdrawal and oral anti-hypertensive regents. The management of surufatinib-related nephrotoxicity without compromising its anticancer effects is challenging. Hypertension and proteinuria must be closely monitored during drug use to reduce or stop the dose in a timely manner before severe nephrotoxicity occurs.

**Supplementary Information:**

The online version contains supplementary material available at 10.1007/s00428-023-03545-2.

## Introduction

Surufatinib is a new, oral, small-molecule TKI that selectively targets VEGFR 1, 2, and 3, FGFR1, and CSF-1R simultaneously [1]. Surufatinib is currently licenced as a monotherapy by the National Medical Products Administration (NMPA) for unresectable locally advanced or metastatic, progressive nonfunctioning, well-differentiated (grade 1 or 2) extrapancreatic and pancreatic neuroendocrine tumours (NETs) [2]. To date, surufatinib is being used for the treatment of solid tumours, including NETs, thyroid cancer, biliary tract carcinoma, and soft tissue sarcoma [3]. As a multitargeting agent, surufatinib seems safe and highly potent, making it one of the most promising targeted therapies. In rare cases, renal adverse events can be observed, including acute kidney injury (AKI) [4] and nephrotic syndrome [1]. Here, we present one case of surufatinib-induced renal thrombotic microangiopathy (TMA) clinically presenting with nephrotic syndrome.

## Case report

**Fig. 1:**
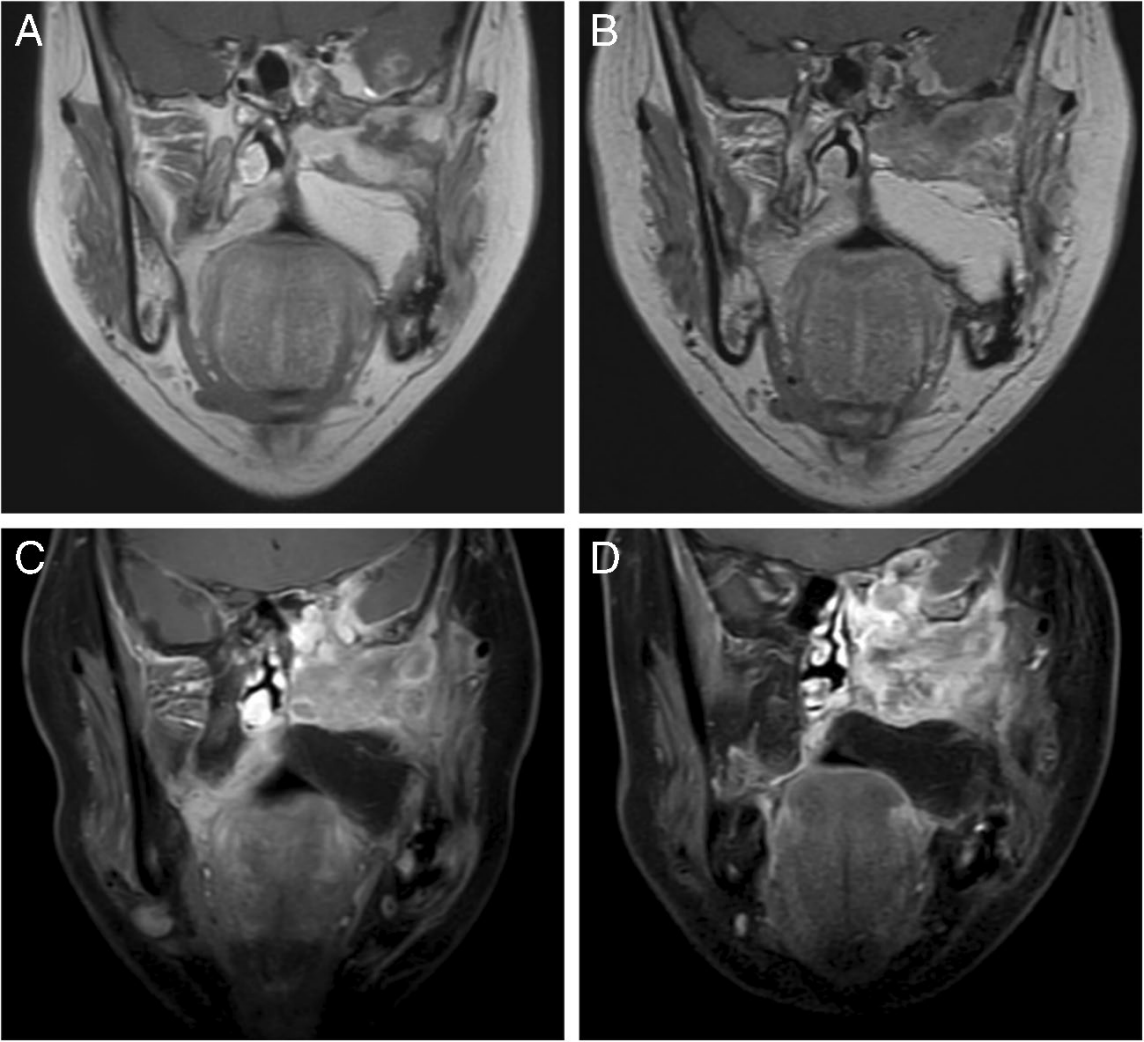
Magnetic resonance imaging (MRI) demonstrated recurrent ACC involving the skull base and intracranial invasion before surufatinib treatment (**A**, July 22, 2021), on the second month of surufatinib treatment (**B**, September 23, 2021; the tumour size was reduced), on the fourth month of surufatinib treatment (C, November 17, 2021; the tumour size was not further reduced), and before admission (**D**, December 21, 2021; the tumour size was evidently larger)

**Fig. 2:**
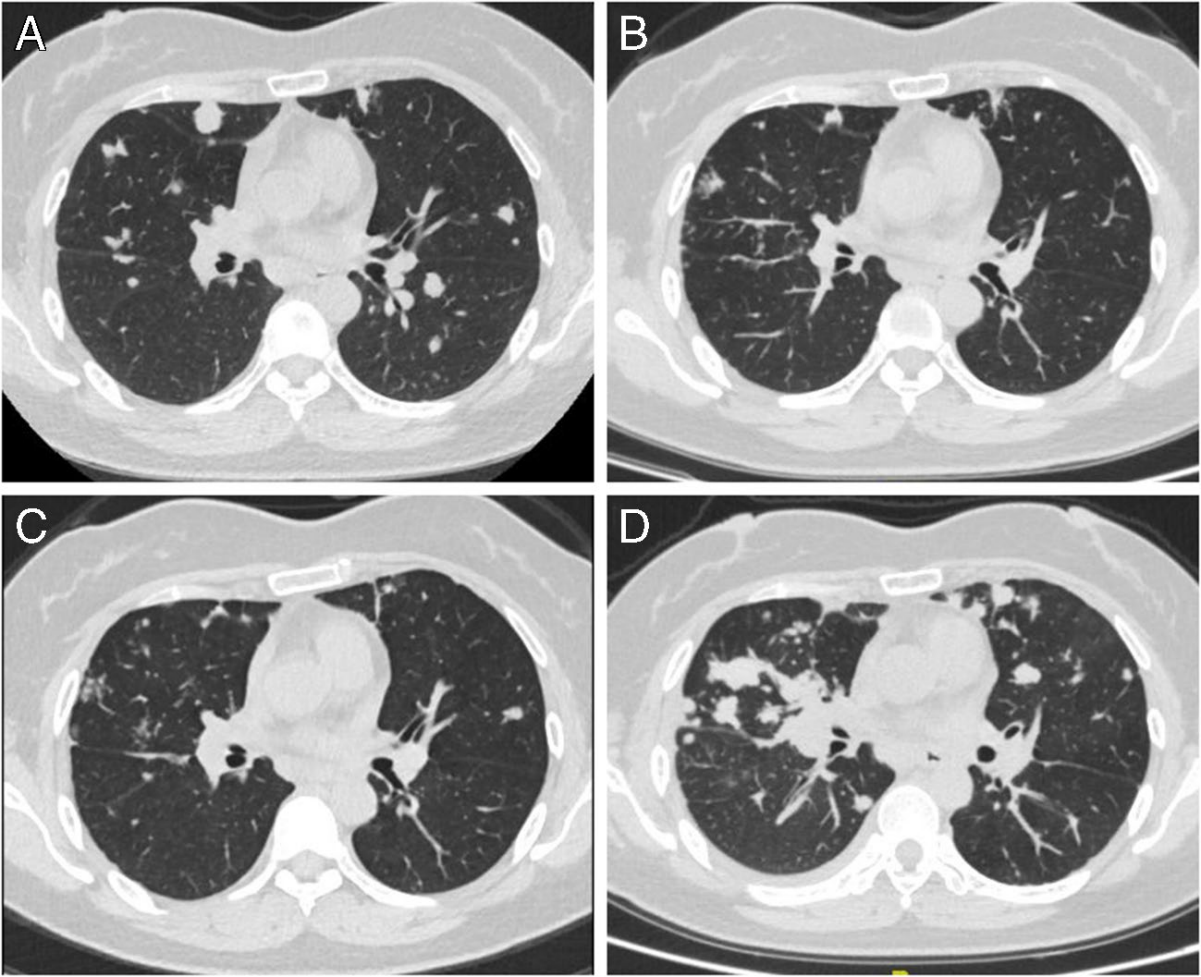
Chest computed tomography (CT) demonstrated lung metastases before surufatinib treatment (**A**, July 22, 2021), on the second month of surufatinib treatment (**B**, September 23, 2021; the tumour size was reduced), on the fourth month of surufatinib treatment (**C**, November 17, 2021; the tumour size was not further reduced), and after surufatinib discontinuation (**D**, February 8, 2022, 49 days from the last dose)

**Fig. 3:**
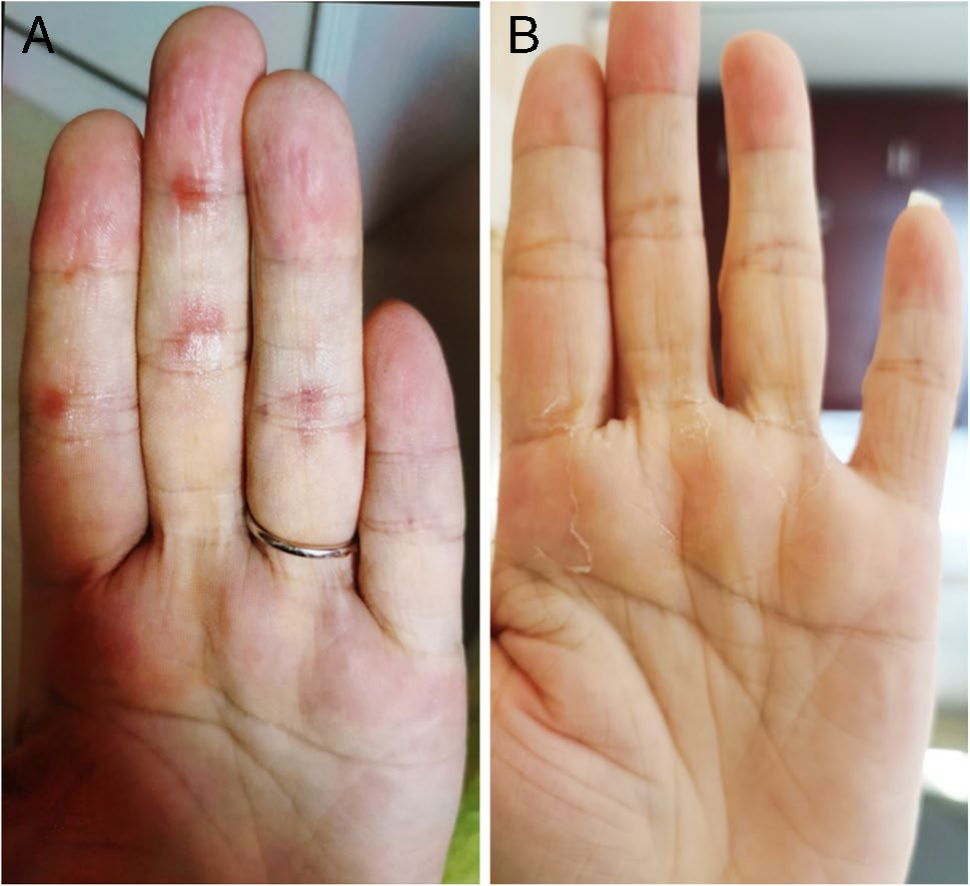
Red rashes developed on the left palm 6 weeks after surufatinib treatment (**A**) and improved after antiallergic treatment (**B**)

The patient fully understood and signed the informed consent form. The patient was a 43-year-old woman who was in good health until August 2020 when she experienced constant pain in the left maxilla. A maxillectomy was performed, and a pathological diagnosis of adenoid cystic carcinoma (ACC) was verified. No treatment was performed after operation. The patient gradually developed pain again in the left maxilla starting in May 2021. When evaluated on July 22, 2021, a recurrent tumour invading the base of the skull and the brain and lungs was documented by magnetic resonance imaging (MRI) (Fig. 1A) and computerized tomography (CT) (Fig. 2A). She was enrolled in a phase II trial with surufatinib for ACC (NCT04910854). The patient received oral surufatinib at a dose of 300 mg/day (once-daily dosing continuously, every 28-day treatment cycle). The curative effect was evaluated every 8 weeks. After 4 weeks of surufatinib treatment, the patient’s blood pressure increased (140/90 mmHg), while her serum creatinine level remained in the normal range. A local rash developed on the right hand at week 6 of surufatinib treatment and was controlled by symptomatic treatment (Fig. 3) when her blood pressure was approximately 160/105 mmHg. The mass of the metastatic tumours in both lungs, skull, and brain was considerably reduced after 2 months (Figs. 1B and 2B). In October 2021, for the first time, the patient underwent a urine stick test that revealed proteinuria of 3+ and haematuria of 1+, accompanied by an increase in creatinine (0.799 mg/dL, range 0.440–0.726) and a decrease in albumin (33.2 g/L, range 35–52). Moreover, progressive oedema of the lower legs was observed. At the 4-month evaluation of the surufatinib curative effect (Figs. 1C and 2C), the patient had stable disease. In December 2021, the level of daily urinary protein loss was found to be in the nephrotic range (12.34 g/24 h). There were no direct or indirect signs of haemolysis, and the platelet count was normal. Subsequently, the patient continued to receive surufatinib treatment except for a 2-day self-suspension until disease progression (Fig. 1D).

**Fig. 4:**
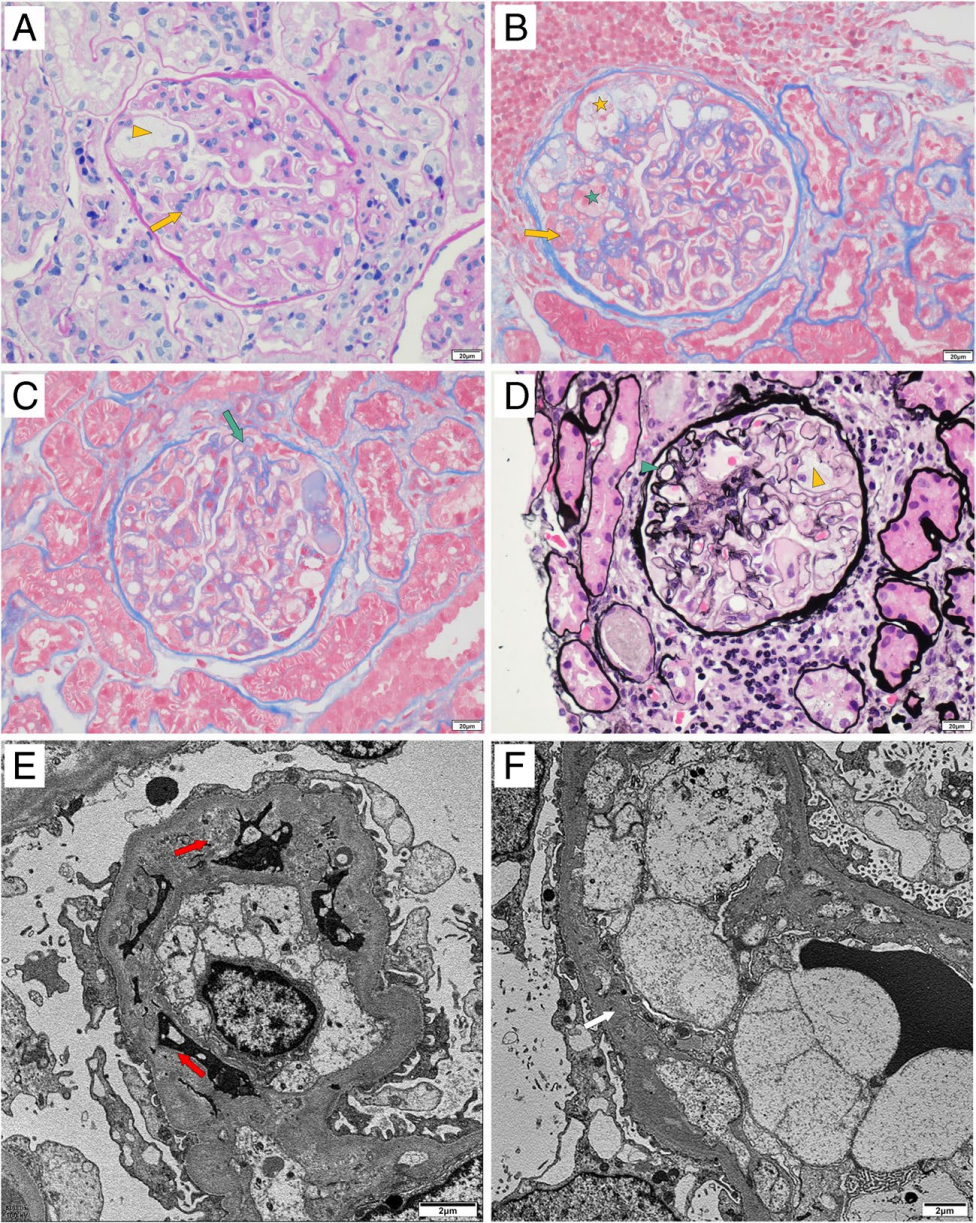
Renal biopsy. Light microscopy (**A**–**D**) shows glomerular endothelial swelling (yellow arrows in **A** and **B**), infiltration of foam cells (yellow arrowhead in **A** and **D**), mesangiolysis (green asterisk in **B**) and dilated capillaries (yellow asterisk in **B**), widening of subendothelial spaces (green arrow in **C**), and double contour (green arrowhead in **D**). **A** Periodic acid-Schiff; **B**, Masson trichrome; **C** Masson trichrome; **D** Periodic acid silver methenamine. Scale bar = 20 μm. Electron microscopy (**E**–**F**) shows double contour of the glomerular basement membrane (red arrows in **E**) and mild subendothelial widening (white arrow in **F**). Scale bar = 2 μm

On admission (December 22, 2021), a physical examination showed a blood pressure of 140/91 mmHg, pulse of 106/min, temperature of 36.3 °C, and respiratory rate of 18/min. As a result of antineoplastic therapy, her body weight increased by 5 to 64 kg. Both lower limbs showed mild oedema. The initial laboratory tests showed the presence of hypoalbuminemia, proteinuria, and hyperlipidaemia (Table 1), leading to the diagnosis of nephrotic syndrome. Kidney biopsy showed glomerular endothelial swelling, mesangiolysis, and double contour, which is consistent with diffuse TMA (Fig. 4A–D). Immunofluorescence showed only weak IgM and faint IgA depositions without any other positive staining (Fig. S1). Electron microscopy revealed double contours of the glomerular basement membrane (Fig. 4E) and mild subendothelial widening (Fig. 4F). The diagnosis was determined to be drug-induced renal damage characterized by endothelial alterations, suggesting a TMA-like pattern.

**Table 1:** Laboratory results at different times

Parameter	2021/8/7	2021/12/22	2022/3/23	Reference range
Haemoglobin (g/L)	143	147	126	105–169; 115–150
White blood cell (× 10^9^/L)	8.9	10.13	6.91	3.50–9.50
Platelets (× 10^9^/L)	176	362	463	125–350
C-reactive protein (mg/L)	ND	4.53	26.33	0–10.00
Creatinine (mg/dL)	0.490	0.869	0.506	0.387–1.320; 0.440–0.726
eGFR (ml/min/1.73 m^2^)	ND	79	117	
Total protein (g/L)	65.2	44	70	65–85; 57–82
Albumin (g/L)	47.9	30	43	40–55; 37–-51
Alanine aminotransferase (U/L)	20.00	14	16	5–40; 10–49
Aspartate transaminase (U/L)	26.00	26	41	5–40; <34
Gamma glutamyl transferase (U/L)	15.10	17	45	7–50; <38
Triglyceride (mmol/L)	ND	4.79	2.32	<1.70
Cholesterol (mmol/L)	ND	7.31	5.35	<5.18
Proteinuria	Negative	3+	ND	Negative
24-Hour proteinuria (g/24 h)	ND	6.21	ND	<0.15
Haematuria	Negative	3+	ND	Negative
Urinary albumin/creatinine ratio (mg/g)	ND	4338.6	1128.3	≤30.0

*eGFR* estimated glomerular filtration rate, *ND* not done

**Fig. 5:**
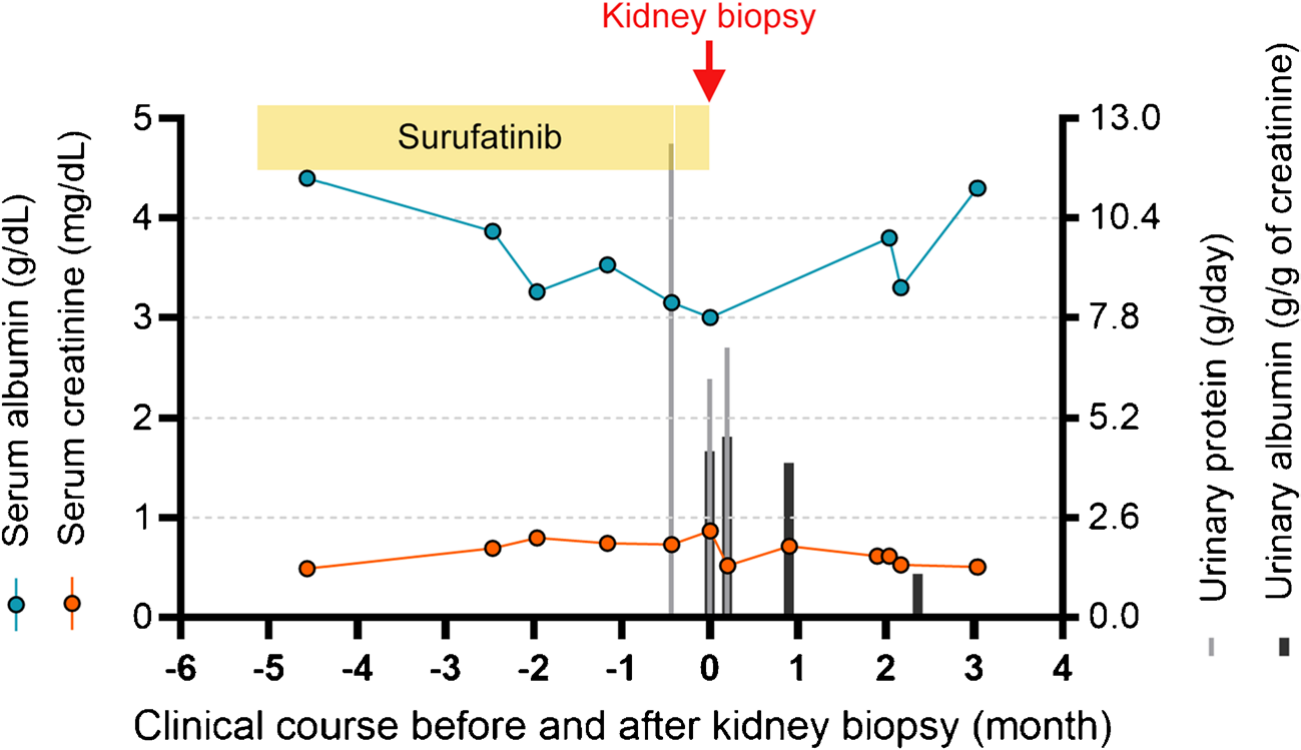
Clinical course before and after kidney biopsy. The period of surufatinib treatment is shown along the top of the figure (yellow rectangle), and the thin white space corresponds to 2-day self- interruption. The month during which renal biopsy was performed (December 24, 2021) was recorded as month 0. Values of serum albumin (g/dL; blue line), serum creatinine (mg/dL; orange line), 24-h urine protein (g/day; grey bars), and urinary albumin (g/g of creatinine; black bars) levels are shown before and after kidney biopsy

Surufatinib was permanently stopped, and anti-hypertensive therapy with valsartan at 160 mg/day was introduced, combined with atorvastatin calcium (20 mg/day) for the treatment of dyslipidaemia. Radiation therapy begun on February 8, 2022, due to the progression of lung metastasis (Fig. 2D). The patient’s renal function gradually improved during follow-up, and by March 23, 2022, her urine albumin level was 1.1 g/gCr, and her creatinine level was 0.506 mg/dL. Because blood pressure and lipids had returned to normal, oral medications were stopped. After radiotherapy, the patient started chemotherapy with nab-paclitaxel, cis-platinum, and cetuximab every 3 weeks starting in May 2022. A follow-up in August 2022 showed that the patient was still alive without any disease-related symptoms and had no abnormal renal function. Fig. 5 provides a timeline overviewing surufatinib management, and the patient’s laboratory results are shown in Table 1.

## Discussion and conclusion

Data from pivotal trials of surufatinib [5, 6] showed an acceptable safety profile in both Chinese and US patients [7, 8], which is similar to other oral angiogenesis inhibitors [9]. All adverse events associated with surufatinib in healthy subjects were grade 1 or 2 [10]. Hypertension and proteinuria, indicating glomerular endothelial damage, were the most common grade 3 or worse treatment-related adverse events (36–38% and 10–19%, respectively) and the most common causes for dose interruption or reduction in only two phase III trials of surufatinib that were completed. However, severe renal damage was not reported in these trials [3, 5, 6]. Among patients receiving surufatinib in six phase 1–2 trials [1, 4, 7, 8, 11, 12], the total incidence of any grade or grade ≥ 3 hypertension was 20.6–63.3% and 2.9–3.3%, respectively; the incidence of any grade or grade ≥ 3 proteinuria was 12.8–90% and 0–14.7%, respectively. Table 2 reports the incidence of any grade and grade ≥ 3 surufatinib-related hypertension and proteinuria in all trials published, which were similar to those reported for other TKIs in multiple clinical trials [13, 14]. However, the incidence of severe renal dysfunction after surufatinib therapy is unclear. Only one case of grade 3 nephrotic syndrome was mentioned in 42 patients with advanced solid tumours [1], and three cases of acute kidney injury were observed in 59 patients with thyroid cancer [4]. However, renal biopsy data have not yet been described. The present case had laboratory evidence consistent with nephrotic syndrome, and the biopsy sample showed histology and electron microscopy findings that were consistent with TMA. To the best of our knowledge, this work reports the histological features of surufatinib-induced renal impairment for the first time. Moreover, surufatinib-related dermatologic reactions, including rash, hand and foot syndrome, and other dermatologic manifestations, were observed only in 7% of patients with advanced NETs in a phase Ib/II trial [7]. Of note, a mild local rash developed on the right hand of our patient, which was likely due to inhibition of the EGFR [15, 16].

**Table 2:** Incidence of any CTCAE grade and grade ≥ 3 surufatinib-related hypertension and proteinuria in completed trials so far

Clinical trials	Disease	Formulation	Subjects	Hypertension		Proteinuria	
				Any grade	Grade ≥ 3	Any grade	Grade ≥ 3
Phase I (NCT02320409) [10]	Healthy	Surufatinib	24	0%	0%	0%	0%
Phase I (NCT02133157) [1]	Solid tumours	Surufatinib	34	20.6%	2.9%	58.8%	14.7%
Phase I (NCT03879057) [12]	Solid tumours	Surufatinib + toripalimab	30	63.3%^#^	20.0%^#^	90%^*^	0%^*^
Phase I/Ib (NCT02549937) [8]	NETs	Surufatinib	39	38.5%	23.1%	12.8%	5.1%
Phase Ib/II (NCT02267967) [7]	NETs	Surufatinib	81	60%	33%	81%	12%
Phase II (NCT02614495) [4]	Thyroid cancer	Surufatinib	59	50.8%	20.3%	72.9%	11.9%
Phase II (NCT02966821) [11]	BTCs	Surufatinib	39	38.5%^#^	17.9%^#^	41.0%^*^	12.8%^*^
Phase III (NCT02589821) [5]	SANET-p	Surufatinib	113	65%	38%	65%	10%
Phase III (NCT02588170) [6]	SANET-ep	Surufatinib	129	64%	36%	68%	19%

^*^Proteinuria included proteinuria and protein urine present

^#^Hypertension included hypertension and blood pressure increased

VEGF-A (also called VEGF)/VEGFR2 signalling is the main driver of tumour angiogenesis and the main target of antiangiogenic therapies, and it also plays a fundamental role in maintaining glomerular endothelial integrity under physiological conditions. VEGF-A inhibitors, either by targeting the ligand VEGF (anti-VEGF) or by inhibiting its receptors (TKIs), have shown remarkable efficacy in improving the prognosis of patients with cancer. Moreover, these drugs lead to glomerular and endothelial cell dysfunction, which is manifested primarily as hypertension, proteinuria, AKI, and renal-specific TMA [17, 18]. Surufatinib is a potent, small-molecule TKI that selectively targets VEGFR 1, 2, and 3, FGFR 1, and CSF-1R, of which VEGFR 2 is mainly responsible for angiogenesis and nephrotoxicity. Abnormal crosstalk between endothelial cells and podocytes mediates TKI-induced nephrotoxicity [19], and glomerular injury might be one of the causes of hypertension [20]; however, the underlying mechanisms are complex and still to be clearly defined.

TMAs are a group of disorders characterized by microangiopathic haemolytic anaemia and thrombocytopenia leading to microvascular occlusion and different levels of end-organ injury [21]. During the last few decades, the incidence of cancer drug-induced TMA has been reported to account for >15% of all TMAs, primarily due to the introduction of VEGF inhibitors [22]. In anti-VEGF agent-related TMA (Type II), nephrotoxicity is characterized by new-onset or exacerbated hypertension, proteinuria (sometimes in the nephrotic range), AKI, and histopathologic features of kidney TMA in glomeruli. Approximately 50% of cases of this type of TMA are limited to the kidney without microangiopathic haemolytic anaemia or thrombocytopenia [23], which may be due to the unique nature of glomerular endothelial cells, which are distinct from the cells in other vessels. To date, TKI-induced TMA, which likely occurs due to the inhibition of the VEGF-A pathway, is rare, and the majority of case reports refer to sunitinib [24]. The typical morphological features of anti-VEGF therapy-induced glomerular microangiopathy include segmental glomerular capillary microaneurysms and segmental hyalinosis, whereas fibrin or platelet thrombi or fragmented erythrocytes are rarely observed or are absent. Individually, these morphological characteristics were found to be accompanied by immune-complex glomerulonephritis [25]. Recent reports suggest that EGFR inhibition is associated with renal disease [26, 27]; however, renal TMA cannot be attributed to EGFR inhibition. Indeed, the subacute TMA that occurred in our patient fit the profile of a VEGF inhibition-induced injury, which was similar to that caused by bevacizumab [27]. Therefore, TMA caused by surufatinib may be mainly caused by VEGF-A inhibition. The analyses of traditionally used biomarkers, such as serum creatinine and blood urea nitrogen, do not provide high sensitivity and specificity or appropriate timeliness for identifying drug-induced kidney damage. The development of novel biomarkers is currently in progress, and metabolomics holds promise for the early and sensitive detection of kidney damage [28]. However, this strategy needs to be confirmed by further studies. Hypertension, proteinuria, and haemorrhage may serve as potential biomarkers of the antitumour efficacy of surufatinib [2]; however, they cannot be used to predict nephrotoxicity due to their variable onset, lack of relationship to dose, and reversibility. Kidney biopsy is one of the most reliable methods used to evaluate renal damage and clarify its cause. An early referral to a nephrology department for evaluation and consideration of renal biopsy is recommended in cases of proteinuria, haematuria, or impaired kidney function during VEGF-A inhibition treatment. The best management strategies to mitigate renal toxicity have yet to be firmly established. Agents blocking the renin-angiotensin-aldosterone system could be preferred due to their added benefit of decreasing proteinuria. Kidney function can usually be improved by combining antihypertensive agents with the reduction or discontinuation of medication. Therefore, collaboration between oncologists, nephrologists, and cardiologists is imperative to prevent and manage renal side effects, which guarantees the best patient outcomes.

In conclusion, we describe the first case of surufatinib-associated renal microangiopathy clinically presenting with nephrotic syndrome. As new TKIs are being developed that directly or indirectly affect the VEGF pathway, TMA is becoming an increasingly important barrier and requires prompt recognition and precise diagnosis. A thorough investigation of the mechanism of VEGF-A/VEGFR2 inhibitor-induced proteinuric nephropathy is the key to resolving this problem.

### Supplementary information

ESM 1: (TIF 9289 kb)
