# Supplementary figures and images for: Surufatinib-induced renal thrombotic microangiopathy: first case report and review of literature
Source: Virchows Arch. 2023 Apr 26;483(4):561–7. doi: 10.1007/s00428-023-03545-2 (PMC10611822; doi:10.1007/s00428-023-03545-2)

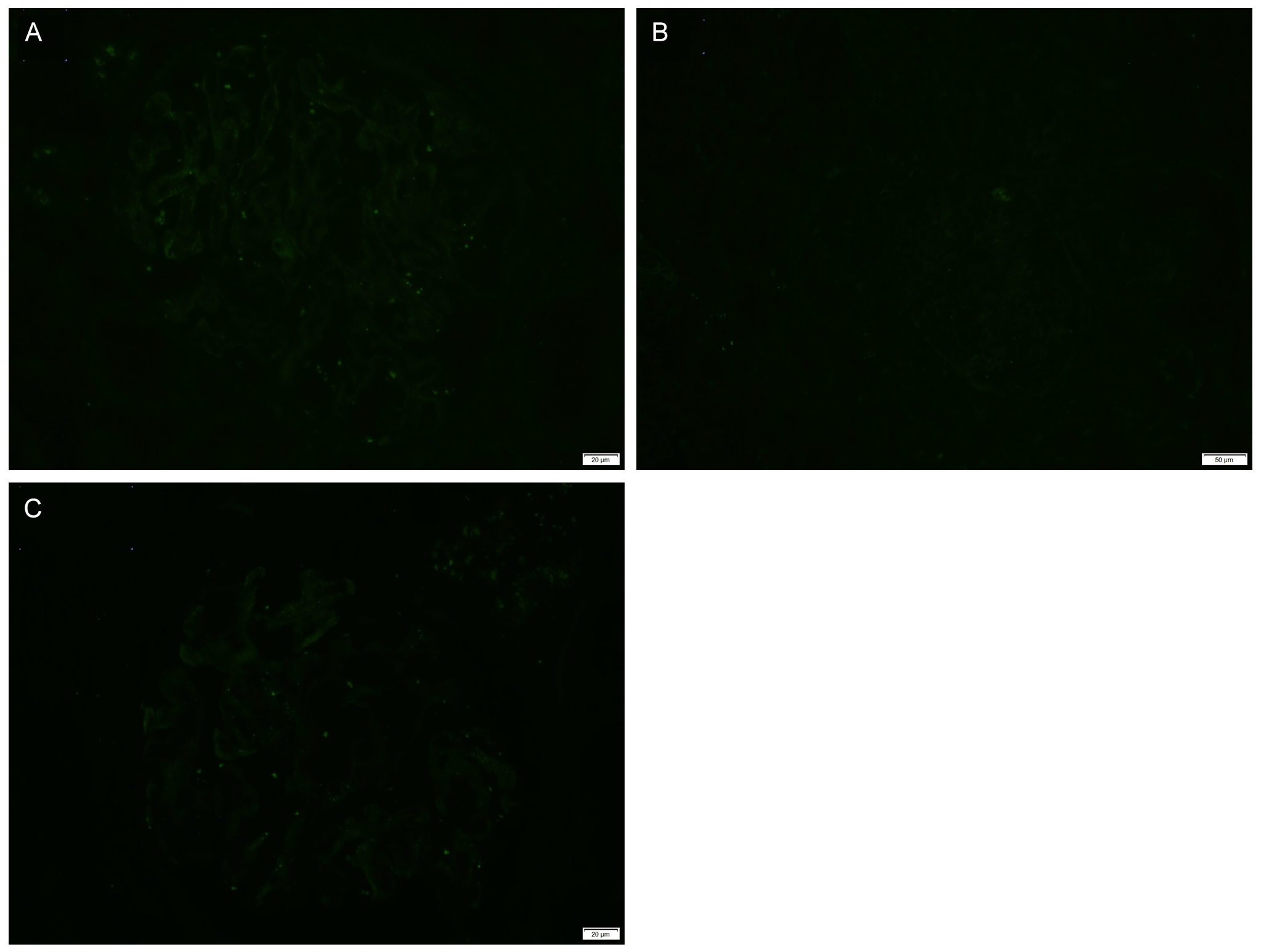

Supplement: Supplementary file 1 — (TIF 9289 kb) [file 428_2023_3545_MOESM1_ESM.tif]
